# Reproductive and Meat Performance of Pekin Ducks (P-11 and P-22) Under a Conservation Programme

**DOI:** 10.3390/ani15111591

**Published:** 2025-05-29

**Authors:** Barbara Biesiada-Drzazga, Eugeniusz Wencek, Dorota Banaszewska

**Affiliations:** 1 Institute of Animal Science and Fisheries, University of Siedlce, Prusa 14, 08-110 Siedlce, Poland; dorota.banaszewska@uws.edu.pl; 2 The National Poultry Counsil—Chamber of Commerce, Naramowicka 144, 60-975 Poznań, Poland; e.wencek@krd-ig.pl

**Keywords:** ducks, reproduction, meat traits, heritability

## Abstract

The aim of this study was to evaluate selected reproductive traits and estimate selected meat traits of male and female ducks of strains P-11 and P-22 during the rearing period, in order to analyse the differences between the two duck populations in terms of these traits in three consecutive generations, i.e., in the years 2020–2022. The birds were kept at the Duck Breeding Centre in Lińsk, Kuyavian-Pomeranian Voivodeship. This study was conducted using all individually tagged individuals of both sexes of known origin and lineage. The birds were kept under standard conditions, and incubation and hatching were carried out in accordance with applicable technology. Reproductive traits were evaluated in three generations of ducks, and meat traits were evaluated during rearing. Ducks of strain P-11 were shown to have a higher laying rate than P-22 ducks, a higher egg fertilization rate, and higher hatching rates of healthy ducklings from set eggs and fertilized eggs, which confirms the differences between the two populations. Average egg fertilization rates during the three-year study period ranged from 92.38% to 94.71% in strain P-11 and from 91.37% to 92.80% in strain P-22. The rates of healthy ducklings hatched from set eggs ranged from 72.24% to 78.15% in strain P-11 and were 2.89% to 4.73% higher than for strain P-22. Egg weight at peak laying (above 80%) ranged from 91.9 to 92.3 g in strain P-22 and from 89.2 to 89 g in strain P-11, and the heritability coefficients for both strains were within the range for moderately heritable traits. Statistically significant differences were shown between the two populations for all meat traits evaluated during the rearing period, i.e., body weight at 3 and 7 weeks of age, keel length, and breast muscle thickness, in each of the three years of this study (three successive generations of birds). Male ducks had higher body weight, better musculature, and higher fat content than females. In addition, the males and females of strain P-22 had higher values for these traits than the birds of strain P-11. Minor changes were observed in successive generations in the values for these traits, as well as in the heritability coefficients estimated from variance for fathers (h^2^_S_), mothers (h^2^_D_), and fathers and mothers (h^2^_SD_). For most meat traits, the heritability coefficients estimated from the variance for fathers (h^2^_S_), mothers (h^2^_D_), and fathers and mothers (h^2^_SD_) took on values from low to high in both strains.

## Simple Summary

Poland has a rich and genetically diverse resource in ducks, which are closely connected to Polish agriculture. In recent years, ducks have become the fastest-growing area of Polish poultry farming, providing highly nutritional and tasty meat. It is Pekin ducks that play the most important role. Research is continually carried out to improve the reproductive and meat traits of these birds, including strains P-11 and P-22. The present study confirmed the high reproductive potential of these ducks and the high biological value of their eggs (egg fertilization, healthy ducklings hatched from set eggs, healthy ducklings hatched from fertilized eggs), with variation in these traits between strains P-11 and P-22. Higher values for laying performance traits and hatching eggs of better biological value were obtained for strain P-11 than for strain P-22. Values for all meat traits were higher in strain P-22, and the differences in comparison to strain P-11 were statistically significant.

## 1. Introduction

The global production of duck meat has been dominated for many years by China, which accounts for 79% of global production, followed by France, Asian countries (Vietnam, Thailand, and Malaysia), the United States, and in Europe, Hungary and Poland. Consumer preferences and favourable market conditions, i.e., lower production costs and a relatively high demand for duck products, are causing a steady increase in the production of these birds in many parts of the world [1,2,3]. Pekin ducks play the most important role worldwide and in Poland [4,5,6,7,8]. Poland has a rich and genetically diverse resource in ducks, which are closely connected to Polish agriculture; in recent years duck production has been the fastest-growing area of Polish poultry farming, providing highly nutritional and tasty meat [7,8,9,10,11,12]. Research is continually carried out to improve the reproductive and meat traits of these birds, including strains P-11 and P-22 [7,13,14,15]. Various methods are employed to this end, including the evaluation of live birds, which makes it possible to estimate selected production parameters. Traditional phenotypic selection and lineage analysis are used as well, followed by the determination of estimated breeding value by the BLUP method [16]. The BLUP (best linear unbiased prediction) method is used in duck breeding to assess the animals’ genetic value. It enables a more precise estimate of genetic value, taking into account data on all known relatives of a given individual as well as various environmental and genetic factors. By taking into account the effect of lineage and other factors, this method enables a more accurate estimate of breeding value. Studies are also conducted to estimate the genetic parameters of egg production in layer ducks [17,18]. For many years, selected breeds and varieties of ducks, including Pekin strains P-11 and P-22, have been covered by a national genetic resources conservation programme in Poland, making it possible to implement numerous breeding concepts while improving existing breeding flocks or creating commercial crossbreeds [19,20]. In addition to maintaining existing genetic variation, a basic goal of the programme is the development of these populations. The programme takes into account the history of the origin of breeds, justifies the protection of individual flocks, and specifies their standards, the aims of the programme, the scope of use value assessment, and the breeding methods. Pekin duck strains P-11 and P-22 were created in the 1970s and are among the oldest populations kept in Poland in terms of the duration of breeding work and the number of generations of ducks that have been evaluated. Both strains are distinguished by very good meat (body weight, thickness of breast muscles, dressing percentage) and reproductive traits (egg weight, egg fertilization) [19,21,22]. Due to their high level of resistance, very high survival rates, and efficient utilization of on-farm feeds, these ducks are predisposed to semi-intensive and extensive rearing. The aim of this study was to assess selected reproductive traits and estimate the meat traits of male and female P-11 and P-22 ducks during rearing, in order to analyse the differences between the two populations in terms of these traits in three consecutive generations.

## 2. Materials and Methods

### 2.1. Study Material

The material for this study comprised male and female ducks of Pekin strains P-11 and P-22 kept at the Duck Breeding Centre in Lińsk, Kuyavian-Pomeranian Voivodeship. All birds were individually tagged. The birds were kept in standard conditions in closed buildings on litter made of rye straw and fed according to nutritional standards. During the laying period, the ducks were fed complete diets containing 16–17% crude protein, 2600–2700 kcal metabolic energy, and up to 7% crude dietary fibre. The birds were reared in groups with an average of 200 males and 700 females for each family. At the age of 23 weeks, 20 flocks of each strain were separated. The birds for these flocks were selected on the basis of conformation corresponding to the Pekin duck standard, the extent of their plumage, and correct posture. During the laying period, small flocks consisting of nine females and one male were kept in separate pens, with individual closed nests in each pen. Incubation and hatching were carried out using standard technology. The flocks were hatched in 2019, 2020, and 2021, and following the rearing period, they were designated for breeding in 2020, 2021, and 2022, respectively. This study included all tagged individuals of both sexes of known origin and lineage.

### 2.2. Research Methods

This study included the monitoring of the laying rate in the duck populations during the first 20 weeks of production, including the number of eggs laid, the number of hatching eggs obtained per duck, and the average egg weight estimated for a period of two weeks at peak laying—laying rate above 80%. The percentage of fertilized eggs and the number of healthy ducklings obtained from set and fertilized eggs were determined.

During the rearing period, the individual body weight of birds was determined at 3 and 7 weeks of age. Zoometric measurements, i.e., keel length and breast muscle thickness, were taken at 7 weeks of age. Based on the data obtained at 7 weeks, the muscle mass and the weight of the fat with the skin were estimated in live ducks. The body weight of each bird was measured using a RADWAG electronic scale with 1 g accuracy. Keel length was measured with a tape measure from the anterior to the posterior edge, to within 1 mm, and breast muscle thickness was measured using the Dramiński 4vet ultrasound machine at a distance of 4 cm from the start of the keel and 1.5 cm laterally from its edge on the left side of the sternum, to within 1 mm.

### 2.3. Statistical Characteristics

In Table 1 the number of hatching eggs per layer during the laying period per initial number of layers are calculated according to the following formula:

number of eggs laid during the laying period

Number of hatching eggs = ------------------------------------------------------------------------ × 100%

number of layers at the beginning of the research period x number of days

The number of hatching eggs per the average number of layers are calculated according to the following formula:

number of eggs laid during the laying period

Number of hatching eggs = ------------------------------------------------------------------------ × 100%

The average number of layers during the research period x number of days

where

the average number of layers stands as the estimated number as a quotient of the sum of layers at the beginning and end of layers divided into two.

The body weight of 7-week-old male and female ducks together with the keel length and breast muscle thickness were used to calculate the muscle mass (Y) and the weight of the fat together with the skin (U), using multiple regression equations [8,23]. The muscle mass and the weight of the fat together with the skin in male and female P-11 and P-22 ducks were calculated using the following equations:

Y = 0.213x1 + 24.760x2 + 62.800x3 − 253.100,

U = 0.247x1 − 32.036x2 + 62.091x3 + 168.369

where

x1—body weight of ducks at 7 weeks of age (g)

x2—keel length of ducks at 7 weeks of age (cm)

x3—breast muscle thickness of ducks at 7 weeks of age (cm)

The muscle mass and the weight of the fat together with the skin, estimated using multiple regression equations, were used to determine their percentage content in the body of each male and female P-11 and P-22 duck. Statistical analysis of the numerical data was performed using SELEKT 1.11 and STATISTICA PL 10.0 software, and means (x), standard deviation (SD), and coefficients of variation (V) were calculated. Analysis of variance was performed for the traits, and the significance of differences was tested by Scheffé’s method. Significance of differences was estimated for a trait determined for a given sex between the two strains at a given age. In addition, the heritability coefficients (h^2^) of the traits were estimated by hierarchical variance analysis from the variance for fathers (h^2^_S_), mothers (h^2^_D_), and fathers and mothers (h^2^_SD_).

## 3. Results and Discussion

Table 1 presents reproductive results for P-11 and P-22 ducks in the years 2020–2022. During the 20-week breeding season, on average for the three-year period, 125.29 eggs were obtained per initial number of P-11 layers and 119.47 from P-22 layers, i.e., about 5.82 more eggs in strain P-11. In both strains, the number of eggs obtained in the first 20 weeks of laying per initial number of layers was highest in 2020—124.4 in strain P-11 and 121.34 in strain P-22. In each of the three years of this study, P-11 ducks had a higher laying rate than P-22 ducks, but the laying rate of both strains can be considered high.

The egg weight at peak laying (above 80%) during the study period was 91.9–92.3 g in strain P-22 and 89.2–89.9 g in strain P-11 (statistically non-significant differences), and the average coefficients of variation (V) were 4.21% and 3.86%. Heritability coefficients for both strains were within the range for moderately heritable traits, amounting to 0.3823 for strain P-22 and 0.3457 for strain P-11. Studies by many authors have indicated the effect of the breed, strain, age, and body weight of layer ducks on egg weight [17,18,24]. The results are supported by other Polish studies on Pekin ducks [25]. Kokoszyński et al. [26] found that Pekin ducks of strain P-44, with higher body weight, laid eggs of markedly lower weight (80.0–86.7 g) than the P-11 and P-22 ducks in the present study. At the same time, the authors indicate that the egg weight markedly increased with the age of the ducks. Many researchers point out the importance of estimating the genetic parameters of egg production, including genetic correlations between body weight and the weight of eggs laid by ducks [17,18]. The results of a meta-analysis by Ghavi Hossein-Zadeh [16] showed that the estimates of the heritability of reproductive traits were moderate, amounting to 0.271 for fertilization rate and 0.238 for hatching rate, and all reproductive traits had low standard deviations.

Average egg fertilization rates during the three-year study period ranged from 91.37% to 92.80% in strain P-22 and from 92.38% to 94.71% in strain P-11. The hatching of healthy ducklings from set eggs in strain P-11 ranged from 72.24% to 78.15%, and these values were 2.89% to 4.73% higher than in strain P-22. The values of these parameters are determined by multiple factors, both genetic [27,28] and environmental [29,30], as well as by the ratio of males to females in the flock [31,32]. In the present study, this ratio was 1:9, which enabled a high egg fertilization rate. It should be added that hatching results from fertilized eggs depend to a very large extent on proper incubation and environmental conditions in the first few days after hatching [2,33,34].

At 7 weeks of age, the average body weight of male P-22 ducks for the period of three generations was 3750.3 g, which was significantly higher than in strain P-11, by 169.9 g (Table 2). The same relationship was found for female ducks of the two strains. The average body weight of 7-week-old female P-22 ducks was 3474.5, which was significantly higher, by 110.4 g, than in strain P-11. In the successive years of this study, a slight increase in body weight was observed in both strains, irrespective of sex. Kokoszyński and Bernacki [19] reported that the average body weight of 7-week-old male P-11 and P-22 ducks was 3257.5 and 3235.0 g, respectively, while that of females was 3097.6 and 2992.1 g. These results are much higher than those obtained earlier by Mazanowski and Książkiewicz [35]. In that study, the average body weight of 7-week-old males was 2293 g for strain P-11 and 2411 g for strain P-22, and the corresponding weights of females were 2249 and 2340 g. In the present study, the heritability coefficients estimated from variance for fathers (h^2^_S_), mothers (h^2^_D_), and fathers and mothers (h^2^_SD_) in both strains of ducks took on values from low to high (Table 2). It can be added here that the results of the meta-analysis by Ghavi Hossein-Zadeh [16] showed that the estimated heritability of meat traits in ducks ranged from low (od 0.156 for carcass weight) to high (0.512 for body weight).

The measurements of live birds throughout the study period (2020–2022) showed that in each of the three years of this study, male and female P-22 ducks had greater keel length and breast muscle thickness than P-11 ducks (statistically confirmed differences). The average keel length in 7-week-old male ducks for the three years of this study was 14.42 cm in strain P-11 and 14.68 cm in strain P-22, and the corresponding values in females were 13.96 and 14.10 cm. The average breast muscle thickness in the live birds of strains P-11 and P-22 was 2.43 and 2.68 cm for males and 2.18 and 2.33 cm for females.

The heritability coefficients for keel length and breast muscle thickness at 7 weeks of age, estimated from variance for fathers (h^2^_S_), mothers (h^2^_D_), and fathers and mothers (h^2^_SD_), took on values from low to high in both strains, with the greatest variation in keel length noted for female P-11 ducks, in which the lowest heritability value was estimated from the paternal component (h^2^_S_) (0.0163) and the highest from the maternal component (h^2^_D_) (0.8192). Low values for heritability coefficients of traits may indicate that their genetic variability is highly variable and thus that further effective improvement of birds by means of mass selection will be difficult. High values, on the other hand, indicate that direct selection can have positive effects.

Based on the estimation of muscle mass and muscle content in live birds, P-11 and P-22 ducks were shown to be well muscled, with the highest average muscle mass noted for P-22 males and females (1076.4 g and 982.2 g, respectively). This was statistically significantly higher than in the male and female ducks of strain P-11 (1019.4 g and 945.8 g, respectively). The estimated content of breast muscles in the body of live birds ranged from 28.0% (P-11 females) to 28.69% (P-22 males). Statistically significant differences for this trait were noted between the strains and the sexes, with significantly higher breast muscle mass recorded for strain P-22. A slight increase in breast muscle content was noted in the final period of this study (in 2022) in both strains. Kokoszyński and Bernacki [19] reported that the average breast muscle content in the carcasses of 7-week-old male ducks was 10.8% in strain P-11 and 11.0% in strain P-22, while the corresponding values for females were 10.7% and 10.3%. In the present study, the heritability coefficients estimated from variance for fathers (h^2^_S_), mothers (h^2^_D_), and fathers and mothers (h^2^_SD_) in both strains of ducks. Muscle mass estimated in live birds at 7 weeks of age took on values from low to high, with the lowest value noted for being heritability influenced by fathers (h^2^_S_) in male ducks of strain P-11 (0.1392), while the highest was estimated from the maternal component (h^2^_D_) in P-11 females (0.8463).

The weight of the fat together with the skin, estimated in live birds on the basis of multiple regression equations, ranged from 666.8 g in P-11 females to 811.5 g in P-22 males. The values for this trait were statistically significant for the strains and sexes of birds. The heritability coefficients estimated from variance for fathers (h^2^_S_), mothers (h^2^_D_), and fathers and mothers (h^2^_SD_) in both strains of ducks for the weight of the fat together with the skin, estimated in live birds at 7 weeks of age, varied from 0.0415 (h^2^_S_) to 0.9034 (h^2^_D_), for P-22 males (Table 2). The estimated content of the fat together with the skin ranged from 20.30% (P-11 females) to 21.09% (P-22 males). Statistically significant differences were confirmed for strains and sexes. Fat content was lower in strain P-11 than in strain P-22 and was also higher in males than in females, irrespective of the strain. As in the case of most of the traits discussed above, the heritability coefficients estimated from variance for fathers (h^2^_S_), mothers (h^2^_D_), and fathers and mothers (h^2^_SD_) in both strains of ducks for the percentage content of fat together with the skin, estimated in live birds at 7 weeks of age, took on values from low to high. Kokoszyński and Bernacki [19] reported higher fat content in the carcasses of female P-11 and P-22 ducks in comparison to males, and at the same time, irrespective of sex, higher fat content in strain P-22, which is in agreement with our findings for the strains.

It should be stressed that in comparison to other livestock species, studies in ducks estimating the indirect and direct effect of mothers and fathers on the production traits of their offspring are relatively scarce [18,36]. Wencek [8] analysed changes in the values of meat traits in 7-week-old P-44 Pekin ducks and reported moderate and high heritability coefficients estimated from variance for fathers (h^2^_S_), mothers (h^2^_D_), and fathers and mothers (h^2^_SD_) for body weight (0.388–0.424), meat weight (0.322–0.453), and fat weight in the carcass (0.322–0.453). Szwaczkowski et al. [37] used several linear models and obtained relatively high estimates for direct heritability (h^2^_a_) for the body weight of strain P-44 Pekin ducks at 3 and 7 weeks of age (0.43–0.45 and 0.53–0.61, respectively) and lower and more varied values for keel length at 7 weeks (0.18–0.27). Indirect heritability (h^2^_m_—maternal heritability) was moderate and did not exceed 0.2 for the traits tested. However, the authors state that the values obtained depended largely on the linear model used.

The results indicate that in each of the three years of this study (three consecutive generations of birds), male ducks had higher body weight, better breast musculature, and higher fat content than females. In addition, P-22 males and females had higher values for these traits than the birds of strain P-11. In successive generations, slight changes were noted for the values of the traits and for the heritability coefficients estimated from variance for fathers (h^2^_S_), mothers (h^2^_D_), and fathers and mothers (h^2^_SD_). 

## 4. Conclusions

The ducks of strain P-11 were shown to have higher laying rates than P-22 ducks, a higher egg fertilization rate, and higher hatching rates of healthy ducklings from set and fertilized eggs. Statistically significant differences in the two populations were noted for all meat traits evaluated during the rearing period, i.e., body weight at 3 and 7 weeks of age, keel length, and breast muscle thickness, in each of the three years of this study (three consecutive generations of birds). Male ducks had higher body weight, better musculature, and higher fat content than females. In addition, the males and females of strain P-22 had higher values for these traits than the birds of strain P-11. Heritability values varied across traits and strains, indicating differing potentials for future genetic improvement.

## Figures and Tables

**Table 1:** Reproductive outcomes of P-11 and P-22 ducks in the years 2020–2022.

	Year—Strain					
Trait	2020		2021		2022	
	P-11	P-22	P-11	P-22	P-11	P-22
Laying rate/initial number of layers (%)	85.65	82.89	84.55	81.06	86.15	81.85
Number of hatching eggs per layer during the laying period - per initial number of layers - per average number of layers	162.83 177.07	157.22 169.64	172.22 187.30	164.61 177.60	122.32 133.02	117.68 126.98
Number of eggs laid per layer up to 20 weeks of production - per initial number of layers - per average number of layers	127.40 138.55	121.34 130.92	124.64 135.55	119.81 129.26	123.84 134.68	117.25 126.51
Egg weight (g) x V SD h^2^_SD_	89.9 3.95 3.55 0.3332	92.3 4.16 3.84 0.3742	89.2 3.77 3.36 0.3582	91.9 4.28 3.93 0.3904	89.6 3.86 3.46 0.3457	92.1 4.19 3.86 0.3823
Deaths and health-related culling during production (%)	3.45	2.83	3.33	2.57	2.42	1.95
Egg fertilization and hatching of healthy ducklings in the breeding flock						
Egg fertilization (%)	92.38	92.25	94.60	92.80	94.71	91.37
Healthy ducklings hatched from set eggs (%)	73.23	70.34	72.24	68.87	78.15	73.42
Healthy ducklings hatched from fertilized eggs (%)	79.27	76.29	76.36	74.21	82.51	80.36

x—mean, V—coefficient of variation, SD—standard deviation, h^2^_SD_—heritability coefficient calculated from the variance for fathers and mothers.

**Table 2:** Means (x), standard deviation (SD), and heritability coefficients estimated from the paternal component (h^2^_S_), maternal component (h^2^_D_), and on average for the paternal and maternal component (h^2^_SD_) of meat traits of male and female P-11 and P-22 ducks during the rearing period in the years 2020–2022.

Trait					Year							
	2020				2021				2022			
					Duck of Strains							
	P-11		P-22		P-11		P-22		P-11		P-22	
					Sex							
	Males	Females	Males	Females	Males	Females	Males	Females	Males	Females	Males	Females
Body weight at 3 weeks of age (g) x SD h^2^_S_ h^2^_D_ h^2^_SD_	1166.86 ^b^ 95.76 0.8720 0.2928 0.5824	1134.78 ^b^ 100.18 0.1599 0.1339 0.1469	1295.76 ^a^ 94.87 0.0570 0.8005 0.4287	1260.21 ^a^ 108.24 0.6702 0.7277 0.6989	1060.0 ^b^ 123.9 0.1130 0.3397 0.2264	1046.5 ^b^ 114.4 0.9765 0.5405 0.7585	1174.4 ^a^ 105.9 0.6345 0.4838 0.5592	1145.1 ^a^ 108.8 0.7851 0.4691 0.6271	1200.60 ^b^ 107.72 0.4508 0.9054 0.7292	1177.74 ^b^ 97.96 0.6168 0.7008 0.6588	1312.03 ^a^ 96.98 0.1623 0.6404 0.4014	1278.57 ^a^ 101.57 0.3157 0.9092 0.6125
Body weight at 7 weeks of age (g) x SD h^2^_S_ h^2^_D_ h^2^_SD_	3579.33 ^b^ 255.74 0.9906 0.2552 0.6229	3351.46 ^b^ 224.47 0.4211 0.7897 0.6054	3787.40 ^a^ 227.26 0.1881 0.5953 0.3917	3491.98 ^a^ 235.71 0.4767 0.7836 0.6302	3467.2 ^b^ 245.4 0.4605 0.6295 0.5450	3283.1 ^b^ 206.1 0.3607 0.6234 0.4921	3621.7 ^a^ 235.6 0.6157 0.5470 0.5813	3408.7 ^a^ 207.9 0.6029 0.7040 0.6535	3694.33 ^b^ 232.35 0.1306 0.8616 0.4961	3457.91 ^b^ 217.45 0.4402 0.6886 0.5644	3841.94 ^a^ 208.24 0.1012 0.8299 0.4656	3522.95 ^a^ 211.45 0.2863 0.9507 0.6185
Keel length at 7 weeks of age (cm) x SD h^2^_S_ h^2^_D_ h^2^_SD_	14.37 ^b^ 0.57 0.9047 0.0369 0.4708	13.96 ^b^ 0.47 0.4703 0.3913 0.4308	14.74 ^a^ 0.52 0.1582 0.4176 0.2879	14.10 ^a^ 0.54 0.4548 0.3267 0.3907	14.3 ^b^ 0.60 0.6146 0.2620 0.4383	13.8 ^b^ 0.50 0.4062 0.3950 0.4006	14.5 ^a^ 0.50 0.6279 0.3265 0.4772	14.0 ^a^ 0.50 0.3104 0.2708 0.2906	14.58 ^b^ 0.496 0.2789 0.7141 0.4965	14.11 ^b^ 0.482 0.0163 0.8192 0.4015	14.81 ^a^ 0.472 0.2797 0.4197 0.3497	14.19 ^a^ 0.489 0.2259 0.5326 0.3793
Breast muscle thickness at 7 weeks of age (cm) x SD h^2^_S_ h^2^_D_ h^2^_SD_	2.43 ^b^ 0.33 0.7516 0.2822 0.5169	2.15 ^b^ 0.30 0.2873 0.8609 0.5741	2.62 ^a^ 0.29 0.2564 0.4241 0.3402	2.31 ^a^ 0.30 0.4621 0.6853 0.5737	2.3 ^b^ 0.40 0.2050 0.5992 0.4021	2.1 ^b^ 0.30 0.1961 0.6642 0.4302	2.7 ^a^ 0.40 0.5873 0.4809 0.5341	2.3 ^a^ 0.40 0.6093 0.4872 0.5482	2.55 ^b^ 0.300 0.0144 0.9987 0.5065	2.29 ^b^ 0.310 0.4496 0.4502 0.4499	2.72 ^a^ 0.267 0.0676 0.8818 0.4747	2.37 ^a^ 0.301 0.2060 0.9947 0.6004
Muscle mass estimated in live birds at 7 weeks of age (g) x SD h^2^_S_ h^2^_D_ h^2^_SD_	1018.40 ^b^ 84.18 0.9182 0.3514 0.6348	941.45 ^b^ 72.03 0.4325 0.8305 0.6315	1083.28 ^a^ 71.96 0.2305 0.5618 0.3961	984.94 ^a^ 75.42 0.5548 0.7684 0.6616	984.7 ^b^ 83.2 0.4097 0.7157 0.5627	919.7 ^b^ 67.8 0.3961 0.5902 0.4931	1043.5 ^a^ 83.0 0.6994 0.5544 0.6269	964.6 ^a^ 72.9 0.5994 0.6899 0.6447	1055.03 ^b^ 72.45 0.1826 0.8056 0.4941	976.33 ^b^ 70.75 0.4491 0.6253 0.5372	1102.41 ^a^ 64.31 0.0889 0.8776 0.4833	997.14 ^a^ 68.11 0.2929 0.9019 0.5974
Muscle content in live birds at 7 weeks of age (%) x SD h^2^_S_ h^2^_D_ h^2^_SD_	28.42 ^b^ 0.42 0.8933 0.4072 0.6503	28.07 ^b^ 0.36 0.4676 0.8463 0.6569	28.60 ^a^ 0.30 0.5423 0.0982 0.3202	28.19 ^a^ 0.35 0.8140 0.4480 0.6310	28.4 ^b^ 0.50 0.4859 0.4815 0.4837	28.0 ^b^ 0.40 0.2335 0.4079 0.3207	28.8 ^a^ 0.50 0.7358 0.3196 0.5277	28.3 ^a^ 0.50 0.5792 0.5169 0.5480	28.55 ^b^ 0.28 0.1392 0.4763 0.3077	28.22 ^b^ 0.34 0.1848 0.8424 0.5136	28.69 ^a^ 0.26 0.6406 0.5214 0.5810	28.29 ^a^ 0.33 0.3458 0.7835 0.5646
Weight of fat with skin in live birds at 7 weeks of age (g) x SD h^2^_S_ h^2^_D_ h^2^_SD_	743.16 ^b^ 73.55 0.8096 0.2823 0.5460	682.75 ^b^ 67.11 0.3494 0.7883 0.5689	794.30 ^a^ 69.30 0.0322 0.7397 0.3859	722.66 ^a^ 69.54 0.3449 0.7248 0.5348	712.1 ^b^ 74.8 0.2067 0.6782 0.4424	666.8 64.1 0.2393 0.7267 0.4830	765.2 ^a^ 77.8 0.5452 0.5081 0.5267	705.4 ^a^ 67.7 0.5883 0.5799 0.5841	772.19 ^b^ 71.39 0.0751 0.9576 0.5164	712.61 ^b^ 67.20 0.4534 0.5411 0.4973	811.49 ^a^ 65.04 0.0415 0.9034 0.4725	730.94 ^a^ 66.61 0.2450 0.9998 0.6224
Content of fat with skin in live birds at 7 weeks of age (%) x SD h^2^_S_ h^2^_D_ h^2^_SD_	20.72 ^b^ 0.73 0.2834 0.1416 0.2125	20.33 ^b^ 0.79 0.2681 0.6392 0.4537	20.94 ^a^ 0.72 0.0645 0.6142 0.3394	20.66 ^a^ 0.79 0.1127 0.4905 0.3016	20.5 ^b^ 0.9 0.1409 0.4799 0.3104	20.3 ^b^ 0.8 0.1317 0.8680 0.4999	21.1 ^a^ 0.9 0.3607 0.4751 0.4179	20.7 ^a^ 0.90 0.4958 0.3190 0.4074	20.87 ^b^ 0.78 0.0751 0.9361 0.5056	20.57 ^b^ 0.80 0.3406 0.4513 0.3960	21.09 ^a^ 0.69 0.0748 0.7699 0.4223	20.71 ^a^ 0.79 0.1892 0.9515 0.5703

a, b—values for a given trait and a given sex between strains differ significantly at *p* ≤ 0.05 (Scheffé’s method). x—mean, SD—standard deviation, h^2^_S_—heritability coefficients estimated from the paternal component, h^2^_D_—maternal component, h^2^_SD_—heritability coefficient calculated from the variance for fathers and mothers.

## Data Availability

All relevant data are included in the article.
